# Supplementary material for: Circulating Inflammatory Cytokine Associated with Poor Prognosis in Moyamoya Disease: A Prospective Cohort Study
Source: J Clin Med. 2023 Jan 19;12(3):823. doi: 10.3390/jcm12030823 (PMC9917516; doi:10.3390/jcm12030823)
Supplement: Supplementary file 1 [file jcm-12-00823-s001.zip › Supplementary table.pdf]

Supplementary Table S1. Differences in IL-6 and TNF-α levels between the diabetic and non-diabetic groups

Variables	N	Levels	ALL Patients (n=204)	No diabetes (n=175)	Diabetes (n=29)	P
IL-6 ,n(%)	204	G1(≤ 2.00)	69(33.824)	65(94.203)	4(5.797)	0.003
		G2(2.00-3.40)	69(33.824)	56(81.159)	13(18.841)	
		G3(3.40-4.83)	33(16.176)	31(93.939)	2(6.061)	
		G4(> 4.83)	33(16.176)	23(69.697)	10(30.303)	
TNF-α ,n(%)	204	G1(≤ 6.24)	75(36.765)	68(90.667)	7(9.333)	0.153
		G2(6.24-8.10)	76(37.255)	64(84.210)	12(15.789)	
		G3(8.10-9.26)	27(13.235)	24(88.889)	3(11.111)	
		G4(> 9.26)	26(12.745)	19(73.076)	7(26.923)	
IL-1β ,n(%)	204	NORMAL(0-5.00)	162(79.412)	139(85.802)	23(14.198)	0.988
		HIGH(> 5.00)	42(20.588)	36(85.714)	6(14.286)	

Supplementary Table S2. Differences in IL-6 and TNF-α levels between the RNF213 mutant and non-mutant groups

Variables	N	Levels	ALL Patients(n=123)	Unmutated (n=130)	Mutated (n=36)	p
IL-6 ,n(%)	166	Normal(0-3.40)	111(66.867)	83(74.775)	28(25.225)	0.116
		High(> 3.40)	55(33.133)	47(85.456)	8(14.545)	
TNF-α ,n(%)	166	Normal(0-8.10)	119(71.687)	88(73.950)	31(26.050)	0.030
		High(> 8.10)	47(28.313)	42(89.362)	5(10.638)	

Supplementary Table S3. Univariate regression analysis of risk factors associated with poor prognosis of moyamoya disease

Variables	Value	N	OR	95%CI	P-value
Age		204	1.081	[1.047,1.116]	＜0.001
Gender					
	male	81			
	female	123	0.775	[0.442,1.360]	0.375
Clinical manifestation					
	Ischemic	143			
	Hemorrhagic	61	1.799	[0.977,3.310]	0.059
RNF213 mutation					
	Unmutated	130			
	mutated	36	0.781	[0.373,1.636]	0.512
Unilateral or bilateral					
	unilateral	25			
	bilateral	179	2.403	[0.987,5.853]	0.054
Image Stage					
	early(suzuki 1-3)	127			
	late(suzuki 4-6)	77	1.194	[0.677,2.105]	0.54
Hyperlipidemia					
	NO	174			
	YES	30	1.571	[0.714,3.456]	0.262
Diabetes					
	NO	175			
	YES	29	1.733	[0.773,3.881]	0.182

Hypertension					
	NO	136			
	YES	68	2.631	[1.435,4.826]	0.002
BMI		204	0.994	[0.936,1.056]	0.856
WBC		204	0.956	[0.844,1.082]	0.473
RBC		204	1.256	[0.745,2.115]	0.392
HGB		204	1.001	[0.987,1.016]	0.85
PLT		204	1.001	[0.997,1.005]	0.51
PCT		204	7.678	[0.075,781.885]	0.388
Hcy		204	0.98	[0.935,1.028]	0.415
CHO		204	0.89	[0.663,1.194]	0.436
TG		204	1.031	[0.779,1.366]	0.829
UA		204	1.003	[1.000,1.007]	0.051
Urea		204	1.075	[0.886,1.305]	0.464
Glu		204	1.125	[0.935,1.353]	0.213
IL-6					
	G1(≤ 2.00)	69			
	G2(2.00-3.40)	69	1.34	[0.684,2.623]	0.393
	G3(3.40-4.83)	33	3.678	[1.491,9.074]	0.005
	G4(> 4.83)	33	1.298	[0.564,2.987]	0.539
TNF-α					
	G1(≤ 6.24)	75			
	G2(6.24-8.10)	76	1.959	[1.025,3.747]	0.042
	G3(8.10-9.26)	27	1.983	[0.814,4.829]	0.132
	G4(> 9.26)	26	2.996	[1.180,7.610]	0.021
IL-8		204	1	[0.995,1.005]	0.965

IL-2R		204	0.998	[0.996,1.001]	0.231
IL-1β					
	NORMAL(0-5.00)	162			
	HIGH(> 5.00)	42	1.1	[0.558,2.170]	0.783
Surgical approach					
	Indirect	85			
	Direct	119	1.167	[0.669,2.038]	0.586
