# Supplementary figures and images for: Circulating Inflammatory Cytokine Associated with Poor Prognosis in Moyamoya Disease: A Prospective Cohort Study
Source: J Clin Med. 2023 Jan 19;12(3):823. doi: 10.3390/jcm12030823 (PMC9917516; doi:10.3390/jcm12030823)

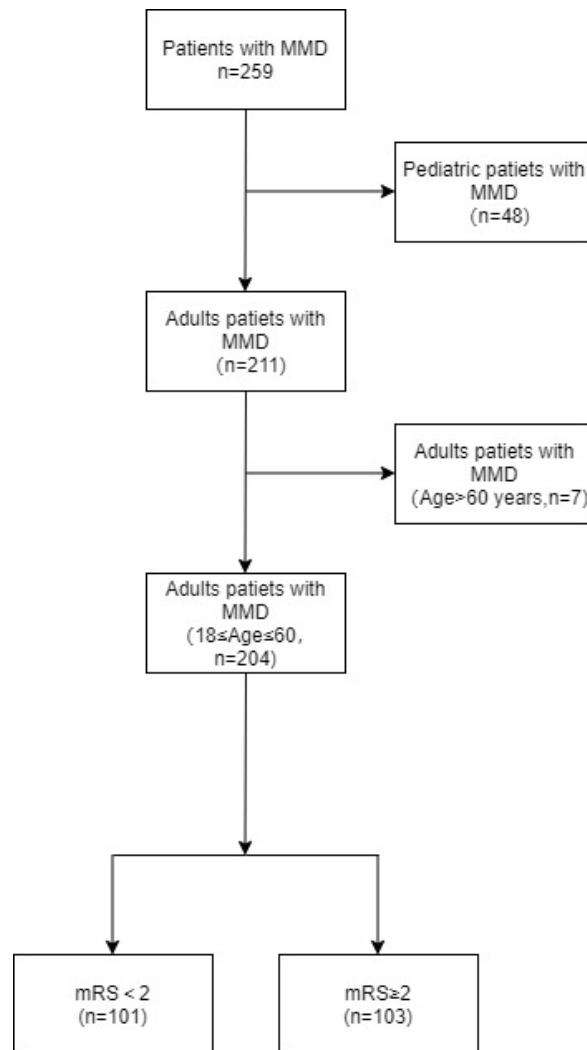

Supplementary Figure S1. Flow chart of the study participants.

Supplement: Supplementary file 1 [file jcm-12-00823-s001.zip › Supplementary figure.pdf]
